# Sizing of airborne particles in an operating room

**DOI:** 10.1371/journal.pone.0249586

**Published:** 2021-04-05

**Authors:** Peter T. Tkacik, Jerry L. Dahlberg, James E. Johnson, James J. Hoth, Rebecca A. Szer, Samuel E. Hellman

**Affiliations:** 1 Department of Mechanical Engineering, University of North Carolina at Charlotte, Charlotte, North Carolina, United States of America; 2 Department of General Surgery, Wake Forest School of Medicine, Winston-Salem, North Carolina, United States of America; 3 West Virginia School of Osteopathic Medicine, Lewisburg, West Virginia, United States of America; 4 Department of Medical Physics, Memorial Sloan Kettering Cancer Center, New York, New York, United States of America; University of Houston, UNITED STATES

## Abstract

Medical procedures that produce aerosolized particles are under great scrutiny due to the recent concerns surrounding the COVID-19 virus and increased risk for nosocomial infections. For example, thoracostomies, tracheotomies and intubations/extubations produce aerosols that can linger in the air. The lingering time is dependent on particle size where, e.g., 500 μm (0.5 mm) particles may quickly fall to the floor, while 1 μm particles may float for extended lengths of time. Here, a method is presented to characterize the size of <40 μm to >600 μm particles resulting from surgery in an operating room (OR). The particles are measured *in-situ* (next to a patient on an operating table) through a 75mm aperture in a ∼400 mm rectangular enclosure with minimal flow restriction. The particles and gasses exiting a patient are vented through an enclosed laser sheet while a camera captures images of the side-scattered light from the entrained particles. A similar optical configuration was described by Anfinrud *et al*.; however, we present here an extended method which provides a calibration method for determining particle size. The use of a laser sheet with side-scattered light provides a large FOV and bright image of the particles; however, the particle image dilation caused by scattering does not allow direct measurement of particle size. The calibration routine presented here is accomplished by measuring fixed particle distribution ranges with a calibrated shadow imaging system and mapping these measurements to the *in-situ* imaging system. The technique used for generating and measuring these particles is described. The result is a three-part process where 1) particles of varying sizes are produced and measured using a calibrated, high-resolution shadow imaging method, 2) the same particle generators are measured with the *in-situ* imaging system, and 3) a correlation mapping is made between the (dilated) laser image size and the measured particle size. Additionally, experimental and operational details of the imaging system are described such as requirements for the enclosure volume, light management, air filtration and control of various laser reflections. Details related to the OR environment and requirements for achieving close proximity to a patient are discussed as well.

## Introduction

In the same way that modifications were made to protocols related to blood borne pathogens at the onset of the AIDS crisis, medical procedures that produce aerosolized particles are under great scrutiny due to the recent concerns surrounding the spread of COVID-19 [1, 2]. Aerosols from chest tube insertion (thoracostomies) and throat tube insertion (tracheotomies), which can range in size from 500 μm particles (which may quickly fall to the floor) down to 1 μm particles (which may float for extended lengths of time), are of interest to surgeons and emergency health personnel. The differences in particle size and quantity expelled during these and other types of procedures affect the potential viral load in the air [3, 4].

Here, we present a method to measure the size of entrained, microscopic fluid particles expelled from a patient during aerosol-generating procedures (AGP). Because the particles are small (high image resolution desired) and the region of interest (ROI) is large (reduced resolution given the same number of pixels), there is an inherent challenge in deploying an imaging solution to cover this large dynamic range [5]. Several standard experimental methods related to particles from the mouth were attempted with marginal or limited applicability [6–9]. The procedure developed for this paper is an extension of a method used by Anfinrud *et al*. and Fischer *et al*. which describe the technique but do not include size calibration method or a method with limited lower measurement resolution [10, 11].

This technique allows quantitative characterization of the size of these particles. The result is a two-part process where 1) particles in a range of sizes are produced and measured using a calibrated, high-resolution shadow method and 2) the same particle generators are measured with the *in-situ*, laser-based shadow imaging system and a correlation mapping is made between the (dilated) laser image size and the measured particle size.

## Comparison of this method to commercial particle sizing methods

Size and velocity measurement of micron-scale particles are typically done in one of three ways. The first employs interferometric methods which are best suited to measuring very small particles (often down to a nanometer). The second are laser-based side scattering methods and are more tuned to very small particle measurement. The third approach are imaging based techniques including the method we are describing in this paper.

### Interferometric particle sizing

Size and velocity measurement of micron-scale particles are commonly done using laser-based interferometric techniques. These vary in configuration; however, they typically measure in the small volume where two or more laser beams intersect one another. This limits the measurement volume to roughly the order of a millimeter (or a volume about that of a grain of rice). There are several commercially available and bespoke interferometric systems based on the phase Doppler principle which perform these measurements. They differ primarily in hardware/software but rely on the same fundamental technique.

Phase Doppler Anemometry or Particle Dynamics Analyzer (PDA), Phase Doppler Particle Analyzer (PDPA), and Phase Doppler Interferometer (PDI) instrumentation systems consists of two laser beams crossing a point in space and a detector measuring the particles where the beams cross [12–15]. Each of the techniques are based on Laser Doppler Velocimetry (LDV)—also sometimes referred to as Laser Doppler Interferometry (LDI) or Laser Doppler Anemometry (LDA)—instrumentation used for measurement of individual particle velocity (and not diameter) by measuring the frequencies of light scattered by individual particles passing through the interferometric laser-beam crossing point [16].

### Sub-micron aerosol instruments

Various commercial instruments exist for measuring sub-micron particles in aerosol applications. These methods have been highly refined, however, the configuration makes them best suited to sub-micron particle measurement and are not generally adaptable for use *in situ*. They would be well suited for the measurement of accuracy in our system (for the smallest particle regime) but are a less cost-effective method and are not capable of a large particle size range. Some commercially available systems include the Scanning Mobility Particle Sizer Spectrometer (SMPS), Fast Particulate Analyzer (DMS500), and Quasi Elastic Light Scattering (QELS) [17–20]. The latter, for example, relies on Rayleigh scattering and the measured particles must be smaller than the wavelength of light use; thus it cannot be used for measuring the size of particles (typically) beyond ∼0.5 *μ*m. Additionally, the measurement volume for each of these tools is limited to a relatively volume and measurements are spatially averaged.

It is important to understand that these are not crude instruments with robust methodologies. Although capable, the use of these methods requires that each technique is tailored to each application. Each application covers a small dynamic range and requires a skilled hand and detailed understanding of the physics to achieve the best possible accuracy of the system.

### Imaging based techniques

Imaging based techniques such as shadow sizing are linear but have a lower boundary to linear size measurement due to pixel size. Nevertheless, their calibration is simple (requiring only a linear scaling) since the particles can be sharply defined.

In contrast, side scatter, structured laser imaging, Structured Laser Illumination Planar Imaging (SLIPI) and other laser based scatter techniques are non-linear but allow a large dynamic range [21]. For the OR system described in this paper, the use of side scatter allows for an advantageous size and orientation of the measurement window. That is, rather than measuring particles travelling past a millimeter sized volume, side scatter measures across a 75mm circle and, rather than measure particles as they speed past a point in space, this system allows the measurement of particles coming towards the detector.

The technique in this paper describes a calibration technique for the non-linear side scatter measurements using a correlation to a shadow measurement. Other techniques use various methods such as light intensity, whereas the method described here uses light dilation or spatial scaling (e.g., imaged particle size).

The cost of this system is relatively low compared with other, commercially-available solutions; It uses a low-cost camera, low-cost particle generators, and a simple dark chamber for the OR. The only high-priced component is the high-speed camera; however, these highly flexible tools are commonly found in research labs.

## Materials and methods

### Calibration: Generation and measurement of particles using shadow imaging method

To calibrate the non-linear side scatter method, particles are generated and sprayed into the OR (side scatter) system and the calibration (shadow) system and a correlation is made between the two systems.

The aerosol particles being studied in the shadow system present two issues with regards to optical measurements; first, they are microscopic, (ranging from < 30 μm to > 500 μm) and second, they are moving quickly, (up to 10 m/s).

Particle size measurements using shadow imaging (back lighting) can be a reliable method which allows for linear calibration/scaling of particle images as well as the measurement of particle velocity; however, the size and speed of the particles during some AGP (in the OR) required the use of a high resolution camera [22] with laser illumination (described below). The laser-based imaging technique used *in situ* is not able to be linearly scaled due to image dilation inherent in side-scattering. Thus, a method of calibrating the laser-based system using a shadow imaging system was developed.

#### Particle generators

**Fig 1:**
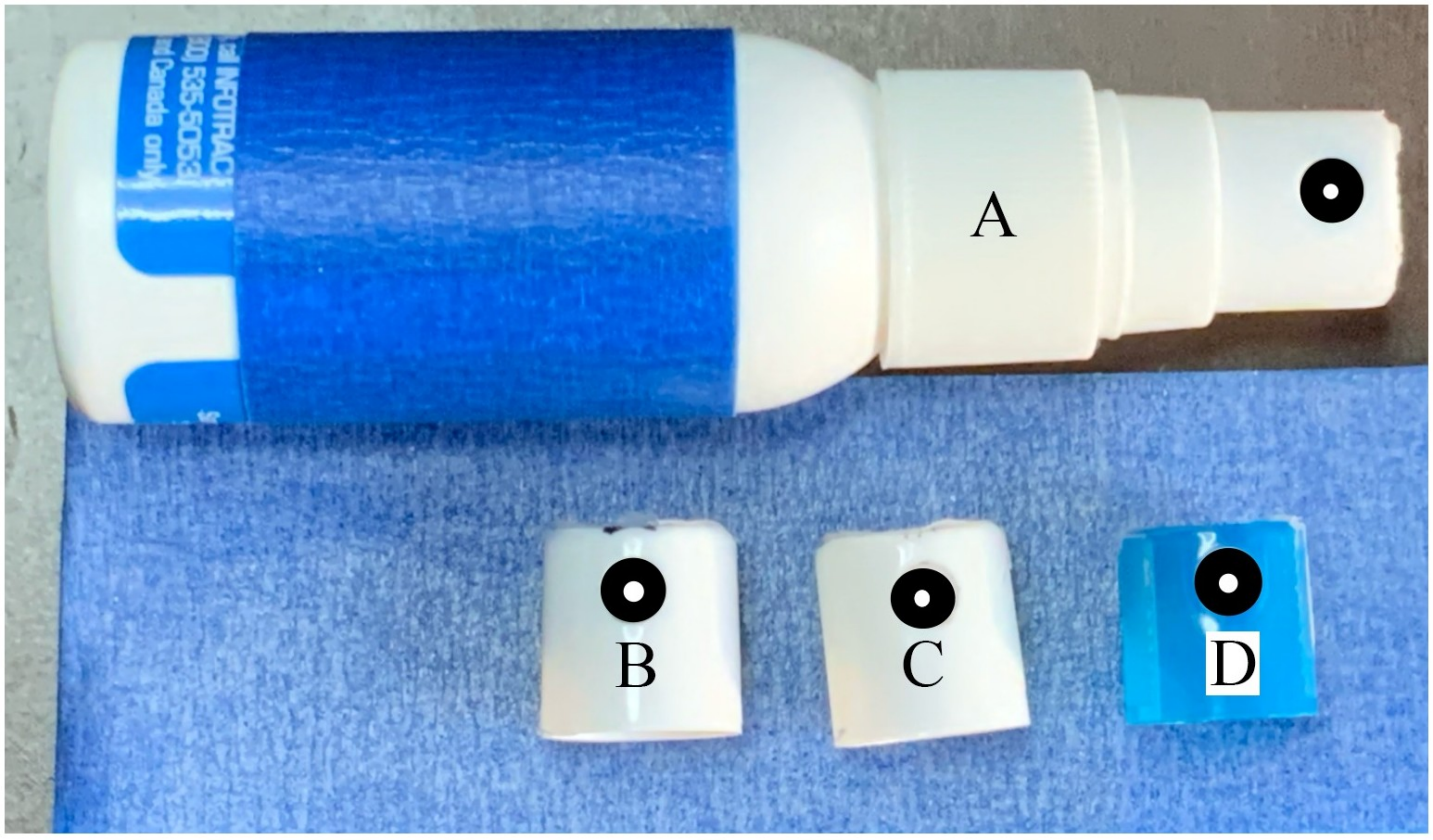
Particle Generator with 0.56 mm orifice nozzle attached and A) 0.74 mm, B) 0.46 mm and C) 1.00 mm orifice nozzles shown.

As shown in Fig 1, modified spray bottle nozzles were used to produce a range of particle size distributions. Using a vibrating orifice generator would also work well and give a single monodisperse size [23, 24], however, the spray bottle solution was chosen due to low cost, flexibility and easy reproducibility. Five particle size ranges were created using nozzles with varying orifice diameters. The standard orifice size of the nozzles was modified by drilling, and the following diameters were used: 0.30 mm, 0.46 mm, 0.56 mm, 0.74 mm, and 1.00 mm. These produced particles with distribution ranges centered between ∼20 μm and ∼650 μm.

While several fluids were tested, water was ultimately chosen due to its minimal environmental concerns and because it most closely matches the luminance of bodily fluids expelled during AGP.

#### Spatial and temporal resolution requirements

To image small particles with a linearly-scaled measurement system, sufficiently high spatial resolution is needed such that each imaged particle occupies more than one pixel. In the shadow imaging (calibration) configuration, one pixel in the object plane represents 20.1 μm in the image plane. To enable this macro focus configuration, a 35 mm lens [25] was used along with a 6.8 mm extension ring. The focus was set at minimum distance and a scaled calibration target (ruler) was positioned in the focal plane (∼70 mm from the lens). The aperture was set to the lens minimum of f/2.8.

In order to acquire enough particle images during each spray event (duration of 0.08 s), an acquisition speed of 10,000 frames per second (fps) was chosen. Due to bandwidth limitations of the camera at 10,000 fps, a cropped ROI of 388 x 344 pixels was used. The spatial resolution of 20.1 μm corresponded with an image plane which was 7.9 mm horizontally and 7.11 mm vertically. At the chosen frame rate and macro scale, the high velocity of the particles required an exposure time of 23 μs to avoid particle streaking in the image.

#### Calibration test protocol for high-speed shadow imaging measurements

**Fig 2:**
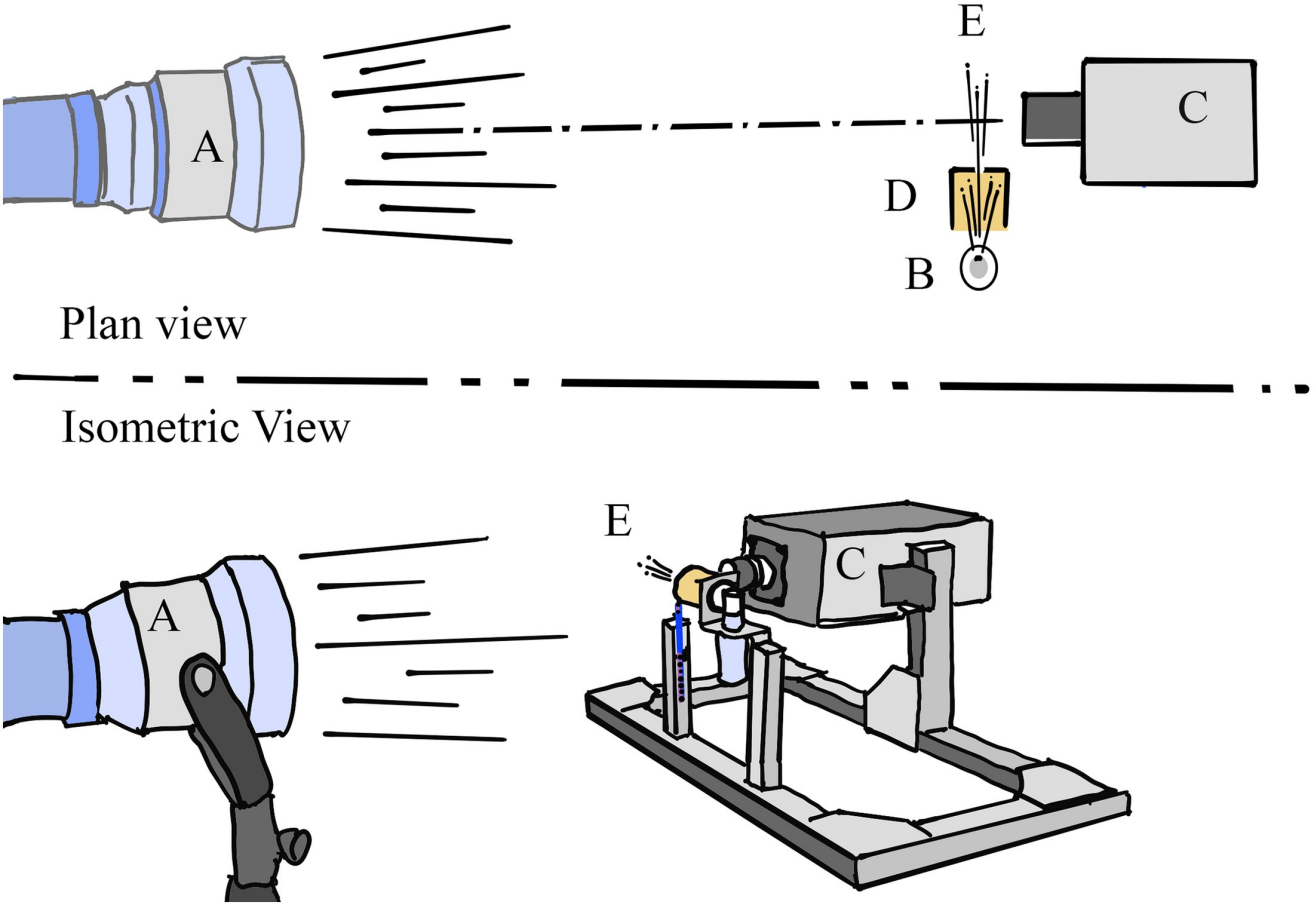
Schematic of the experimental set up from the plan view (top) and isometric view (bottom) showing the placement of A) high-intensity light, B) particle generator, C) high speed camera D) particle shield and E) generated particles.

The exposure time of 23 μs required the use of a high-intensity light source [26, 27]. When this lamp is set at maximum power and shining directly into the camera lens from less than a meter away (see Fig 2), the imaging sensor can easily become overheated. In order to prevent sensor damage, we used a test protocol as follows:

1. Set the camera to record in loop mode onto circular buffer (6 s loop time).
2. Quickly rotate the lamp onto the FOV.
3. Send pre trigger to the camera to start recorded loop.
4. Generate the particles, spraying them across the FOV. Duration of approximately 0.08 s.
5. Immediately turn the lamp away from the camera—typically less than the full six seconds of exposures were acquired.

#### Shadow method calibration FOV

**Fig 3:**
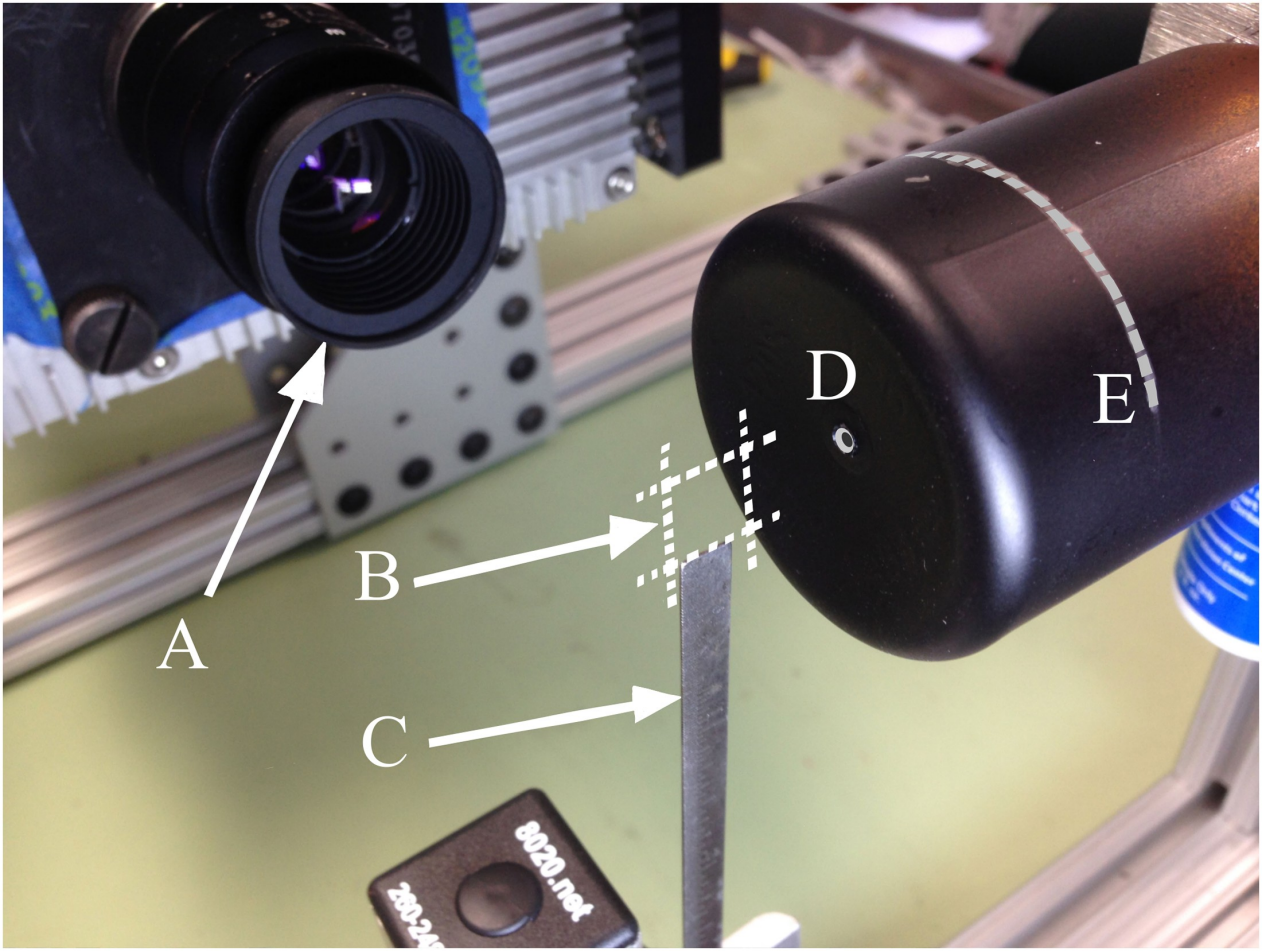
Experimental set up showing A) camera lens, B) insertion reference line on particle shield, C) 3 mm aperture in particle shield, D) 7.9 x 7.1 mm field of view and E) steel ruler.

For the calibration imaging configuration, the FOV was approximately 7.9 x 7.1 mm in the image plane (Fig 3). To position the setup repeatably, a metal frame was constructed that supported the camera, spray nozzle and a translating fixture to position the calibration ruler— 6 in (152.4 mm) x 3/16 in (4.76 mm) with 1/64 in (0.4 mm) resolution [28]—in the FOV. The rigidity of the frame allowed for fine adjustment of focus which could subsequently be locked on the lens.

**Fig 4:**
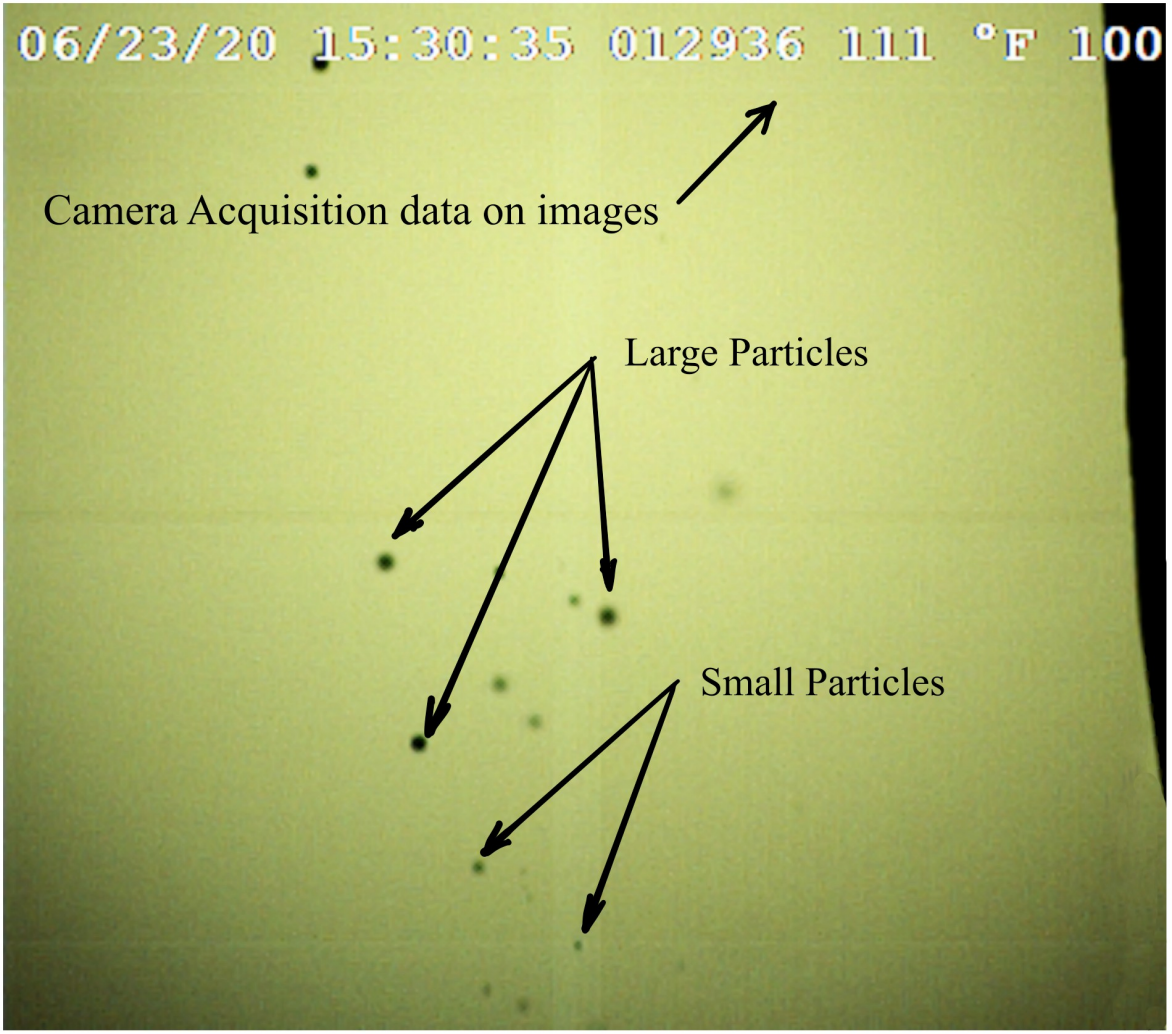
Example image of the particles from 0.74 mm nozzle; 10,000 fps, 23 μs exposure.

Due to the small FOV, it was easy to lose track of the image plane position in space. To address this issue, the ruler was positioned such that, after calibration images were acquired, it could be accurately shifted in the image plane with a small portion remaining visible in the FOV during recording. The dark edge in the upper right corner of the images (e.g. Fig 4) is the tip of the ruler. The ruler also assisted by providing a reference location towards which the particles could be sprayed during testing.

#### Shadow method calibration Depth Of Field (DOF)

**Fig 5:**
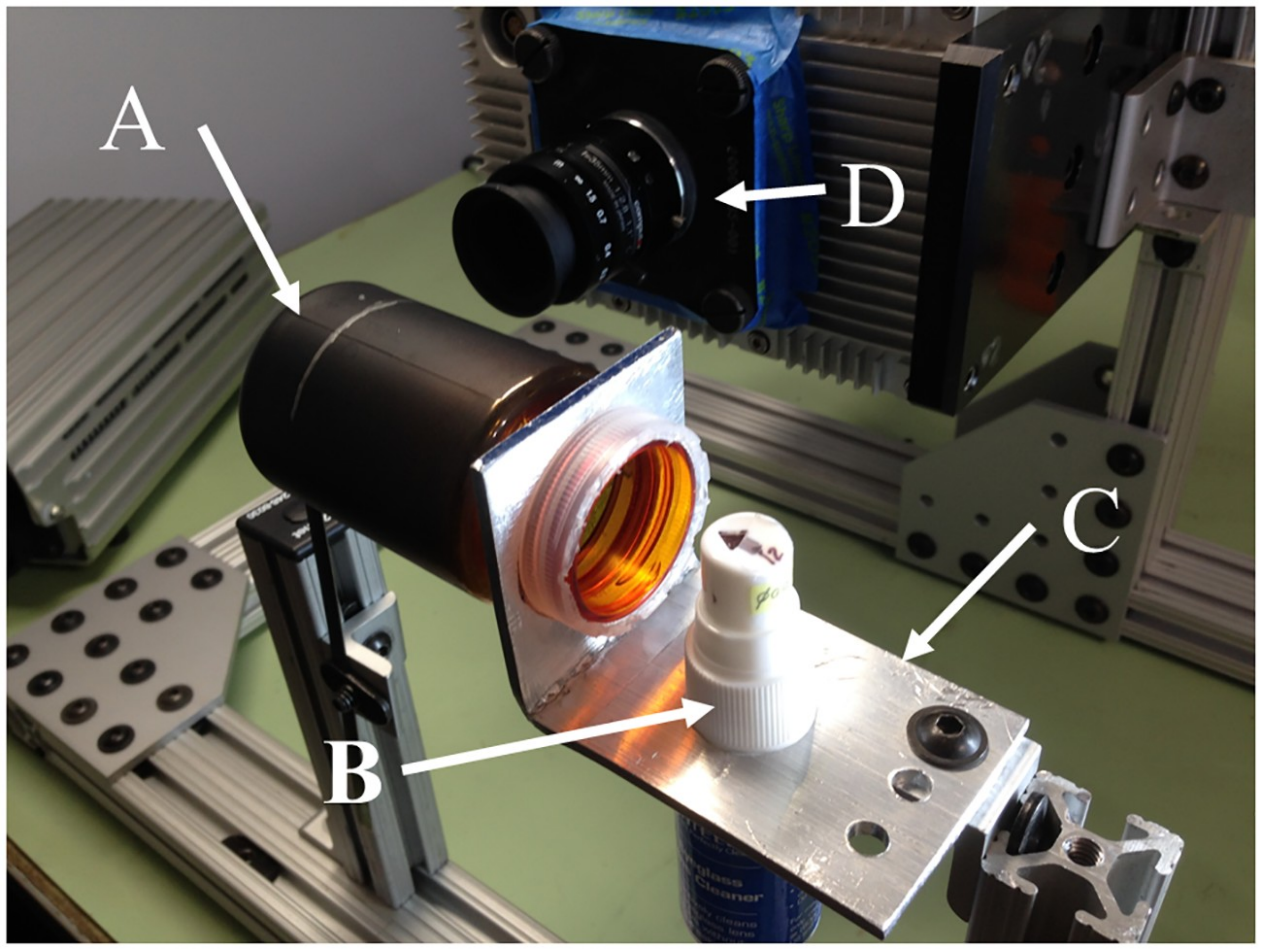
Experimental set up including A) Particle Shield, B) 0.030mm Particle Generator, C) Particle Shield Aluminum Bracket and D) High-Speed Camera.

Due to the large aperture of the lens (f/2.8) required for acquisition with a relatively short exposure time, the resulting DOF was only a few millimeters which could result in imaging many particles outside of the focal plane. In order to reduce the number of out-of-focus particles, a shield was used to limit the location of the particles to this shallow DOF. The shield (Figs 3 and 5) was made from a 50 mm (= diameter) x 70 mm long plastic cylinder with one end open and the other restricted by a 3 mm orifice. The particles were sprayed into the shield and only exited through the 3 mm aperture on the far end. The cylinder was painted flat black to minimize laser reflections.

An aluminum bracket was fabricated to hold and align the particle generators (sprayers) with the shield and the bracket was mounted to the calibration frame. A silver reference line was drawn around the circumference as a visual aid for repeatable insertion depth into the OR system as seen in Figs 3 and 5.

#### Shadow method calibrated particle size measurement

The high-speed camera [29] images were acquired using the manufacturer’s supplied image capture software [30]. This system aquires the high-speed images as a collection of single TIFF format frames. Each calibration test resulted in 6,000 recorded frames, of which only those with particles visible in the FOV were stored (between 80 to 120 frames per experiment). These image sets were acquired in technical replicates for each of the five orifice sizes, (0.30, 0.46, 0.56, 0.74, and 1.0 mm). Fig 4 shows an example of a single image gathered during one of the tests with a 0.74 mm orifice.

The same software was also used to play back the sequences where each particle size, position, and velocity were manually measured. Particle velocity was determined by tracking the position across successive frames while size was determined by measuring the particle diameter in the image (in pixels) and multiplying by the 20.1 *μ*m/pixel scale factor. The standard deviation of the velocity was used as a data recording check, (i.e., the range of standard deviation was about 0.15 to 0.20 and an input error would typically result in a value over 100).

#### Out-of-focus particles

While the previously described shield limited the number of out-of-focus particles, there were still some captured in the calibration images. To minimize the error of diameter measurement due to blurry particles, two methods were employed. First, only reasonably clear particles (qualitatively assessed) were measured.

Secondly, a diameter estimation technique was developed with a calculation made to determine the effective diameter of a marginally blurry particle. An ROI of approximately twice the estimated particle size was selected around each particle to be analyzed, (e.g., 16 x 19 pixels for a 10.4 pixel diameter particle and 8 x 10 for a 4.3 pixel diameter particle). The gray values were normalized by the average background (bright) value and the pixels were then inverted such that each pixel was calculated by using Eq 1,

$$\begin{array}{c} G_{e}={\left(\frac{G}{G_{b}}\right)}^{-1} \end{array}$$ (1)

where *G*_*e*_ is the effective pixel gray value, G is the captured gray value and *G*_*b*_ is the average background gray value. For an ideal shadow image with perfect focus/contrast, this would result in a gray value of 1 for all pixels inside the imaged particle and 0 for all background pixels. In actual captured images—and particularly for blurred images—a gradient is present. The calculated diameter was determined by summing all resulting gray values in the ROI and treating this sum as the effective particle area [in pixels]. From this effective area, the diameter was calculated assuming a circular particle with Eq 2,

$$\begin{array}{c} A_{e}=\frac{\pi *d_{e}^{2}}{4} \end{array}$$ (2)

where *A*_*e*_ is the effective area and *d*_*e*_ is the effective diameter. The effective diameter [in pixels] was then calculated using Eq 3,

$$\begin{array}{c} d_{e}=\sqrt{\frac{A_{e}}{4*\pi }} \end{array}$$ (3)

and converted to *μ*m using the scale factor of 20.1 *μ*m/pixel.

Qualitatively assessing the edge determination on several examples indicated that the calculated diameter of a marginally out-of-focus particle was a good representation of the physical boundary. The calculated edge was approximately halfway through the perimeter gradient of the imaged spot; midway between the dark central shadow and the bright background level.

#### Results of high-speed shadow measurements

**Fig 6:**
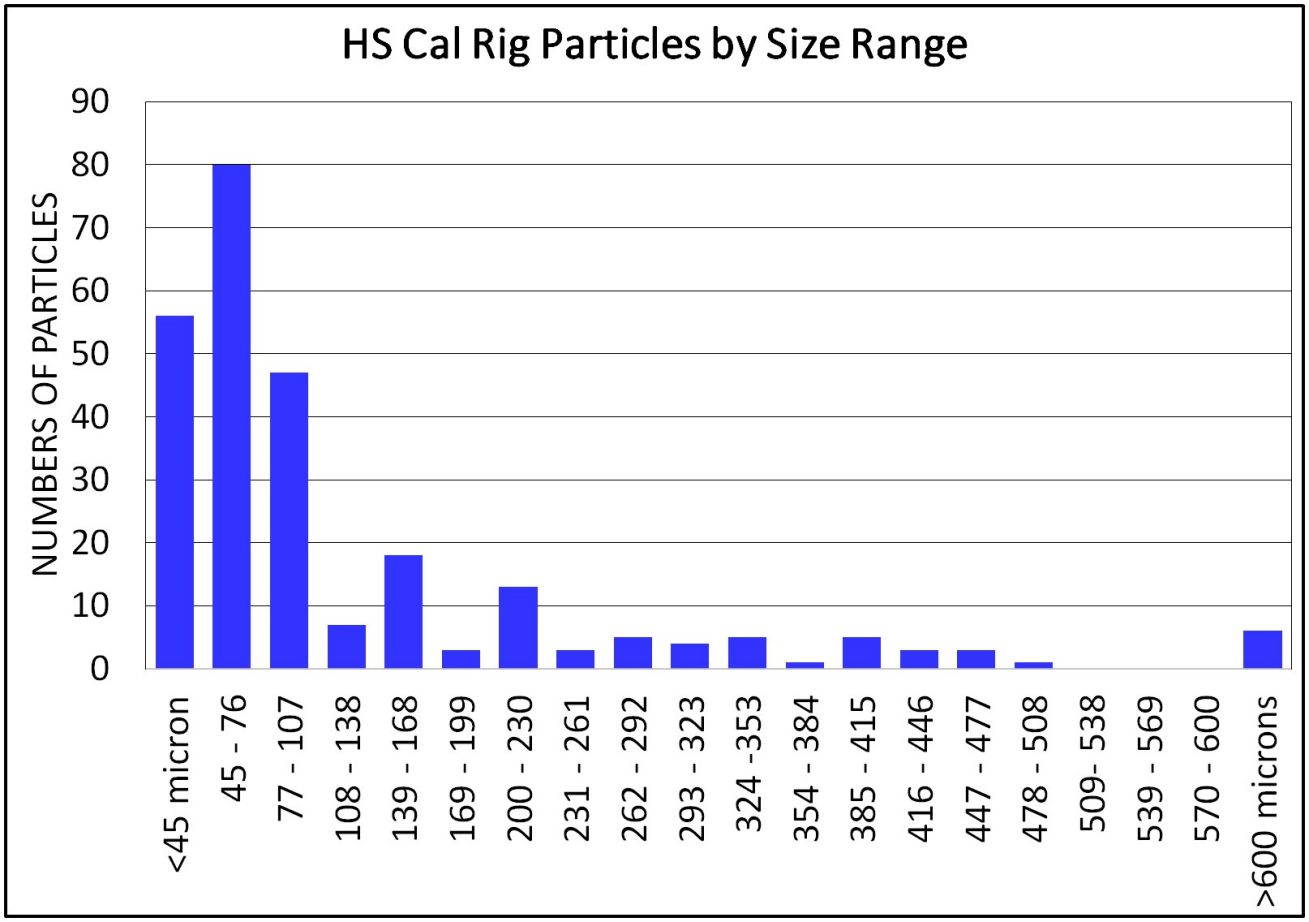
Histogram of particles counts in high speed shadow method by size.

In total, 260 particles were measured. Each nozzle was tested in technical replicates and within each experimental run, every particle was measured multiple times (average 5 times) in successive image frames as it traversed across the FOV. Measurements from all five particle generators—including replicates—were combined for subsequent analysis. Fig 6 shows the distribution of measured particles.

### In-situ measurements with OR system: Laser-based imaging in mobile enclosure

The generation and measurement of particles using the shadow imaging method described in the calibration section yielded the reference data needed for calibration of the in-situ, OR measurement system (“OR System”). For measurements in an OR environment, a mobile system was developed with a non-intrusive FOV ten times larger (∼75 mm square versus ∼7.9 mm square) than the calibration configuration. The mobile system can be moved easily to the OR and the combined system weight for the aluminum frame, enclosure, camera, laser, etc. is 14 kg. The OR System includes a dark enclosure designed to allow recording of the side-scattered laser light from particles produced by the same particle generators detailed in the previous calibration section. Repeating measurements on the same particle generators with both systems allowed us to correlate the imaged particles diameters from scattered laser light in the OR System to the particle sizes measured in the calibration system.

#### Aluminum support frame

For the OR environment, the two most critical concerns are safety (for health care practitioners and patients) and repeatable placement of the test equipment. Both concerns are primarily related to the control and positioning of the laser system. The laser must be a) in a fixed position with its beam and reflections contained and b) rigidly aligned with respect to the camera. To this end, an adjustable aluminum frame was fabricated to support the entire system. The frame is symmetric such that the laser and camera can be reversed to allow work on either side of the patient [31].

### Laser with sheet optics

The laser used produced a 5000 mw continuous laser in green 532Nm light [31]. Sheet optics spread this to a tall but thin sheet of laser light of about 120mm x 2mm at the entrance aperture of the light containment enclosure. The controls were set at 50% power during testing both in the lab and the OR. Locking linear sleeve bearings were attached to the bottom of the laser to allow quick attachment to the aluminum frame for both left, and right configurations. Mechanical stops were adjusted on the aluminum support frame so that the laser beam position could be repeatedly achieved on the left and right side with little need for adjustment.

#### Light containment enclosure

**Fig 7:**
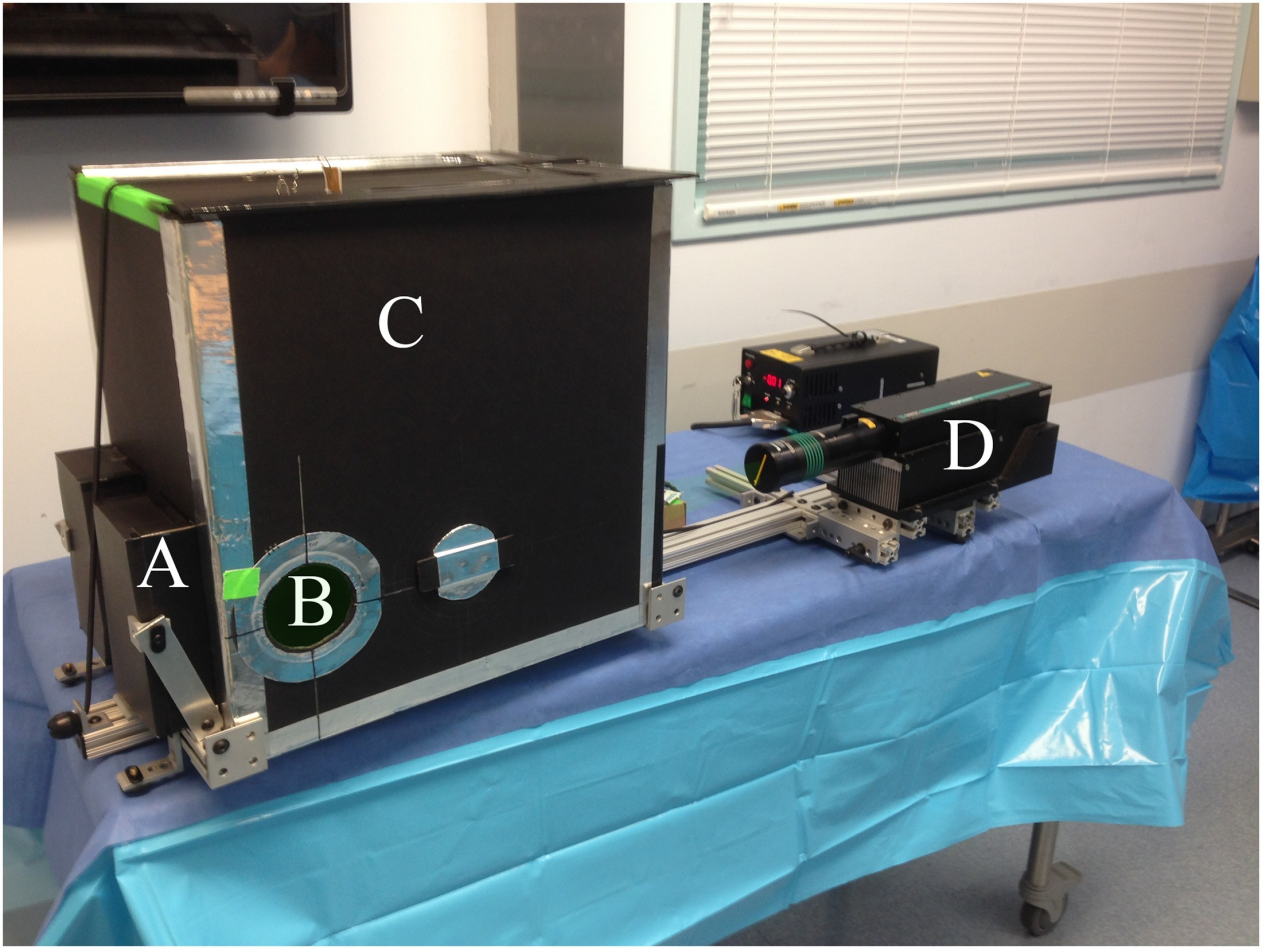
OR system in left orientation with A) laser beam traps, B) circular intake aperture, C) dark chamber, and D) laser.

**Fig 8:**
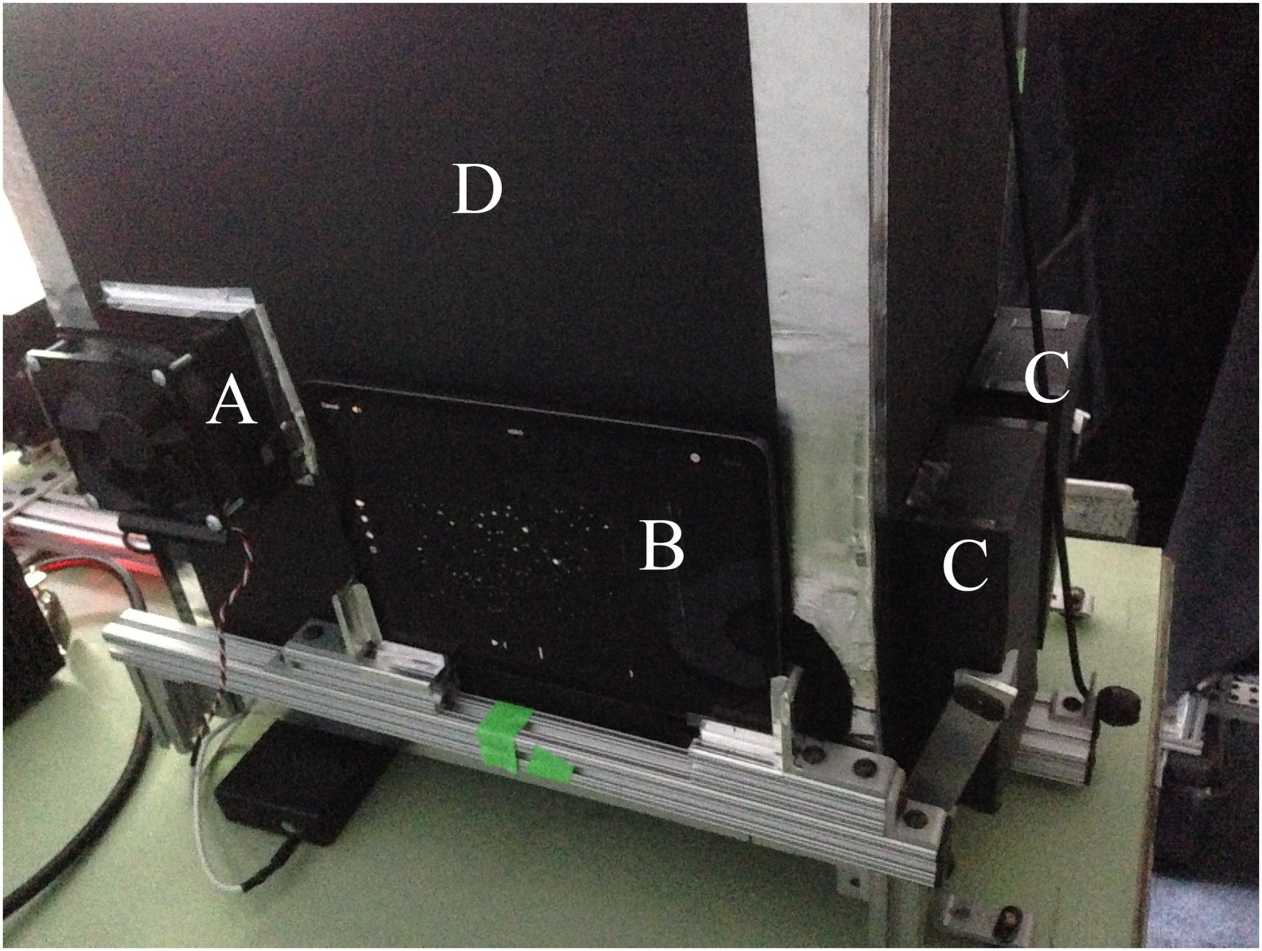
Rear of enclosure showing A) fan and filter, B)tablet with support frame, C) left and right laser beam traps and D) dark chamber.

**Fig 9:**
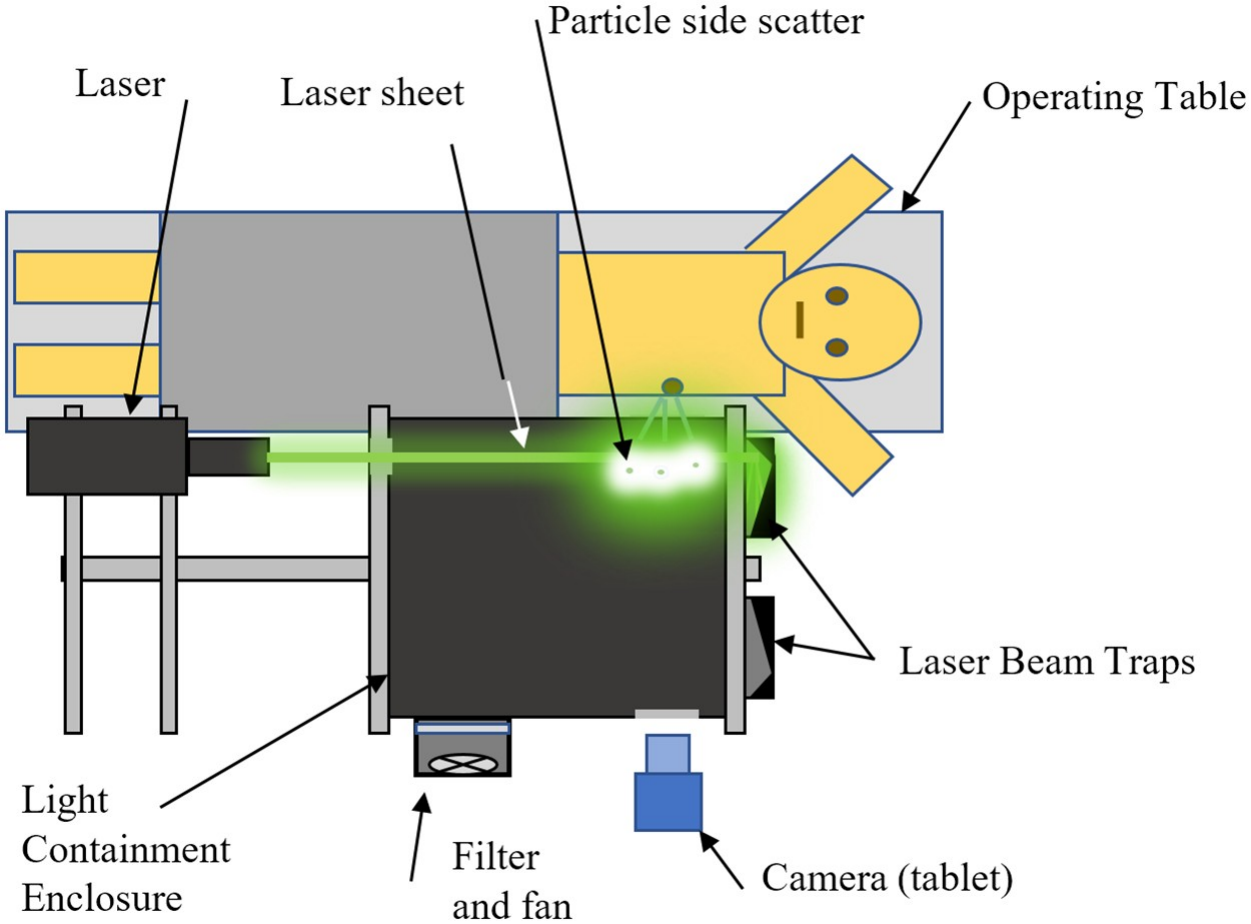
Overhead schematic of OR system in left thorocotomy /thoracastomy orientation.

The OR System as seen in Figs 7–9 includes a dark enclosure which contains the particle flow from the patient along with the laser light and reflections. There are apertures for the particles exiting the patient as well as to allow access for the camera and laser. It is constructed of black foam board (6 mm thick) and has outer dimensions of 457 x 457 x 305 mm with a volume of 63.7 liters; smaller enclosures were found to cause flow restrictions. To minimize weight, the aluminum frame supports the enclosure at its lower outside corners and foam board structure is held in place with an elastic rubber cord. This allows the break-down for transport, quick access to the inside and simple replacement. Since the frame does not fully surround the enclosure, this also allows simple and flexible modification for different surgical procedures, anatomical locations and levels of access; the foam board can be easily cut with a craft knife (e.g., X-Acto) prior to surgery to increase access. In the most extreme case, the whole front and much of the bottom could be cut away.

While the authors created a flexible system design which can accommodate many procedures, the enclosure in the present study was configured for lateral thoracic access (required for, e.g., thoracotomy/thoracostomy). In this configuration, the enclosure box sits on the patient’s side and is fit as close as possible to the axilla with its long edge roughly parallel to the abdomen. There are two apertures directly opposite one another on the lateral sides of the enclosure close to the proximal (superior) end (near the axilla). One aperture (entrance aperture) is positioned around the incision site to allow particles from the patient to enter the enclosure, while the opposite aperture (camera aperture) allows the camera to view the particles. The camera looks directly across the enclosure at the entrance aperture. Laser light enters through a slit in the distal (inferior)end of the enclosure normal to the viewing axis of the camera. The entrance aperture has a light “friction-fit” removeable ring which allows it to be changed from 75 mm to 120 mm, although most measurements were made with the 75 mm ring.

#### Laser beam traps

To minimize laser reflections, black cardboard beam trap boxes are attached to the enclosure and held in place by diagonal braces (see Fig 7). These boxes are 150 mm tall and have a 15 mm wide vertical entrance slit. There are two boxes to allow use for configurations with the laser on either side. After the slit, an internal diagonal board reflects the light sideways into a chamber which has a narrow converging section. Despite the intensity of the laser light, these beam trap boxes minimized reflected light well and, even during a 45-minute trial, did not overheat.

#### Filtered air

**Fig 10:**
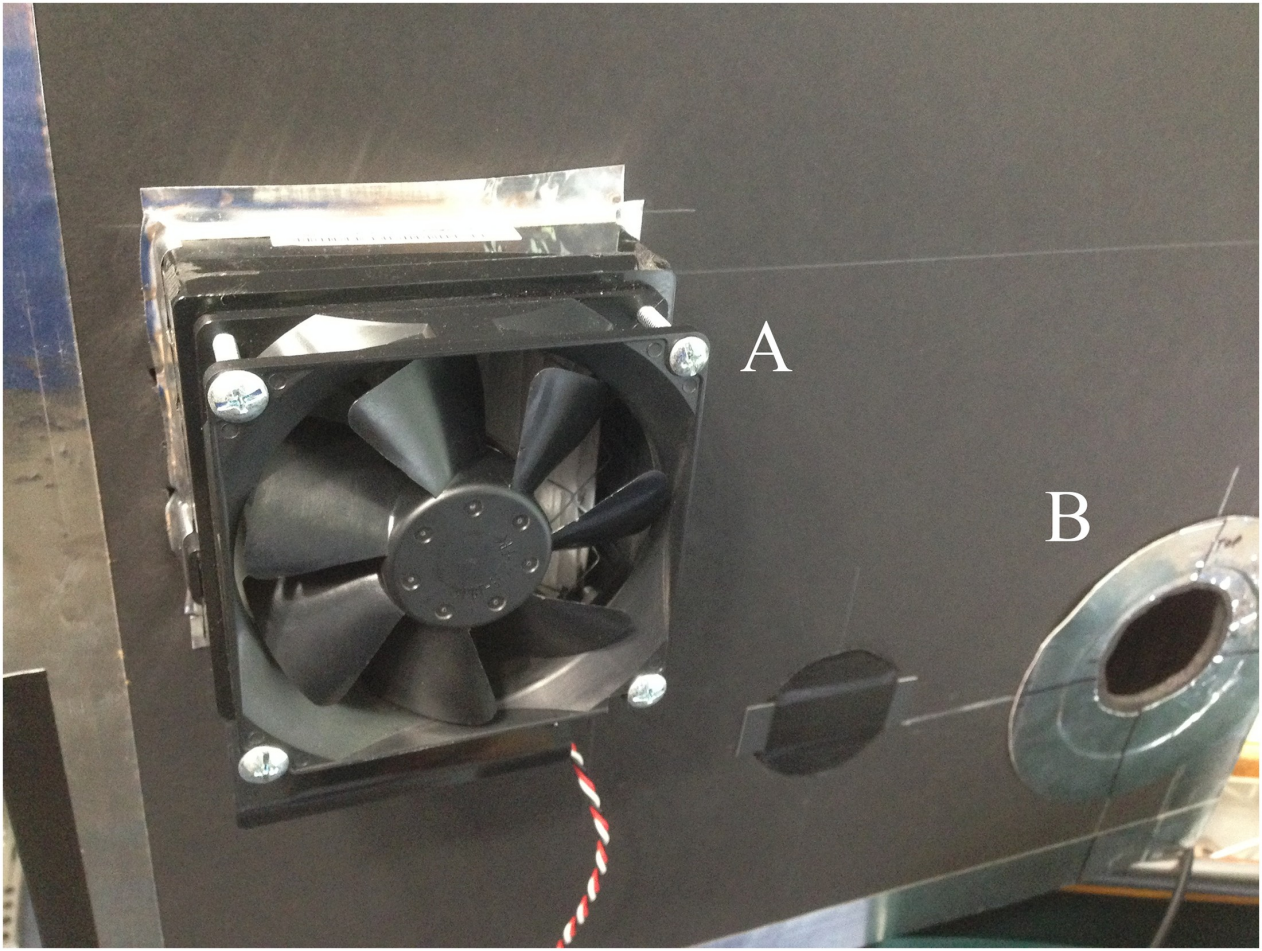
Right side of enclosure with A) filter and fan, and B)entrance aperture.

A 100 mm (Fig 10) square fan with air filter is installed on the upper left side of the enclosure to maintain positive pressure with clean air and minimize noise in the data from airborne dust. It is used in the OR but needs to be turned off during experiments as it is powerful enough to reduce particle flow into the enclosure. It is constructed from a common brushless DC motor (a.k.a. “muffin”) fan, blows into the enclosure through a HEPA air filter, and is powered by a 12 VDC battery.

#### Camera

The experiments were recorded with a computer tablet [22] set to acquire video in 4K-60 mode to achieve video with a resolution of 3840 x 2160 pixels at 60 fps. The tablet is mounted in a removeable (left or right side) support frame against the camera aperture located directly across from the entrance aperture of the enclosure. As described in the light containment enclosure section, the camera views across the enclosure directly into the entrance aperture to record particles coming from either the particle generator or patient exhaust gas.

To focus the camera on the desired FOV, a 20 mm square micro-detailed focus target was clipped to the entrance aperture and positioned in the plane of the laser sheet. A flashlight was then directed onto the target from the camera aperture. With the target illuminated, the image of the focus target was pressed until the tablet focused on the target. Once focused on the target, both the focus and the exposure were locked (AEAF Lock).

#### OR system particle generator measurements

To map the particle diameter measurements from the shadow imaging calibration data, the same particle generators were measured in the enclosure of the OR System. The particle generators and nozzles described in the particle generators section (including shield) were measured in the OR System. To acquire the test video, the following procedure was followed.

1. Turn on the laser and let it warm up to full power.
2. Run the air filter fan for at least 30 seconds.
3. Insert focus target.
4. Illuminate focus target.
5. Focus camera and lock the camera settings.
6. Remove focus target.
7. Turn off fan.
8. Start recording video.
9. Insert and spray the particle generator several times.
10. Stop recording

**Fig 11:**
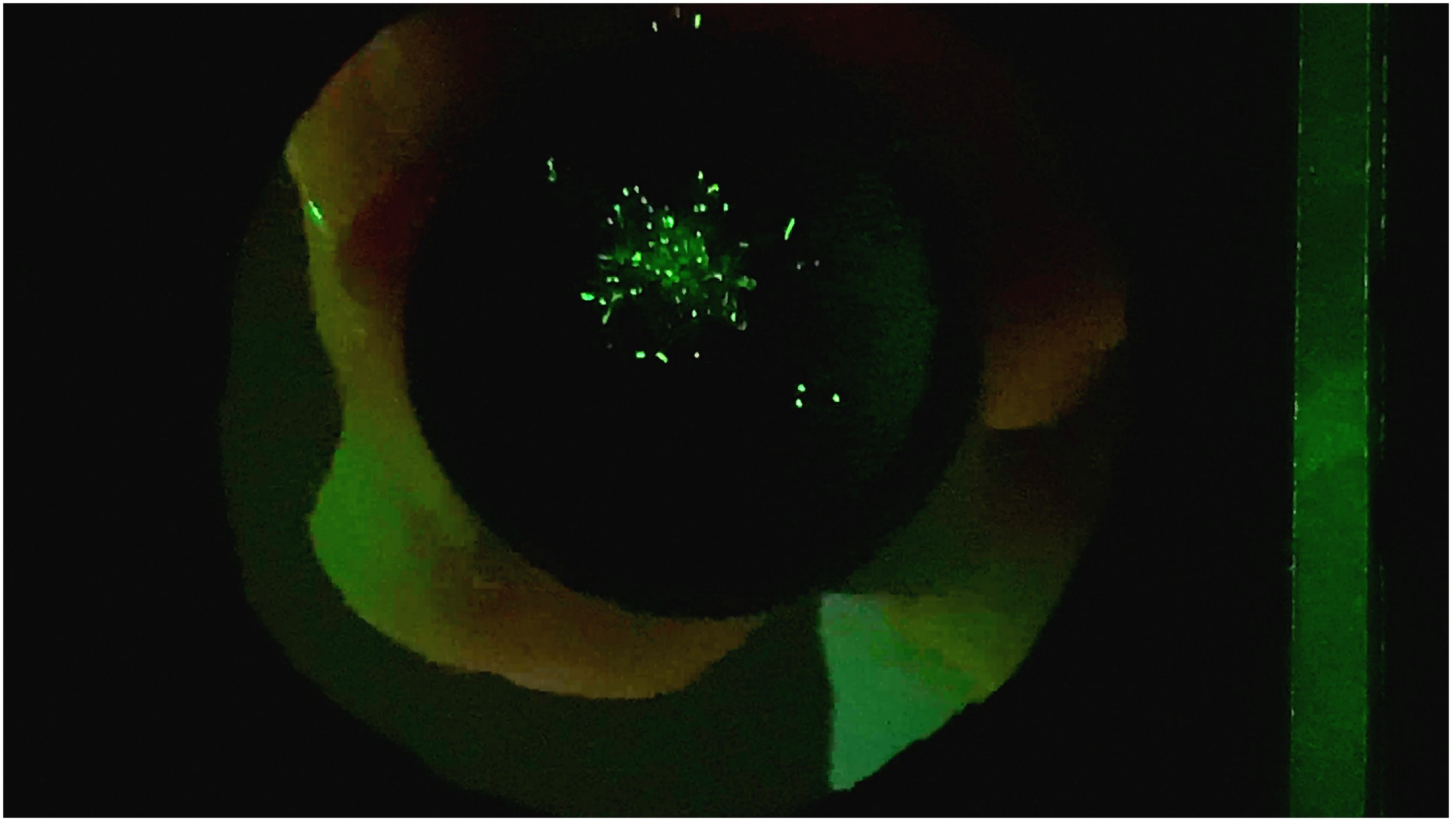
Single image frame from video of 0.46 mm particle generator experiment showing scattered laser light from particles.

Each video was converted to a string of single TIFF images (sample image shown in Fig 11). From the five particle generator nozzles (with technical replicates for each size), 1123 particles were measured. For the OR System, the camera frame rate is lower and, thus, most particles were only measured once during each experiment since they did not generally appear in more than one frame of the video. In several cases, repeat measurements were possible, however, transverse velocity data was not used due to this limitation. The effective diameters of the particles were determined using the same technique described in the calibrated particle size measurement and out-of-focus particles sections, without the last step of converting from pixels to physical units. This resulted in an effective diameter [in pixels] for each particle measured.

#### Correlation of mobile OR system and shadow calibration measurements

Since the same particle generators were used along with the same number of repeat measurements for each size, it is assumed that the distribution of particle sizes should be similar between the shadow imaging calibration data and the data measured in the OR System. However, the total number/magnitude of measured particle counts did not match between the two systems due to a) large difference in field of view and b) differences in system sensitivity. In order correlate the two histograms’ distributions, the OR System test measurement data counts were normalized to the calibration data using a constant scale factor. The two distributions were then parsed into varying numbers of bins in order to converge on a best fit to the calibration data. Additionally, overflow and underflow bins were used due to linear resolution limits of the OR System (all particles above and below the linear resolution limits appear to be the same sizes in a scattered-light configuration).

**Fig 12:**
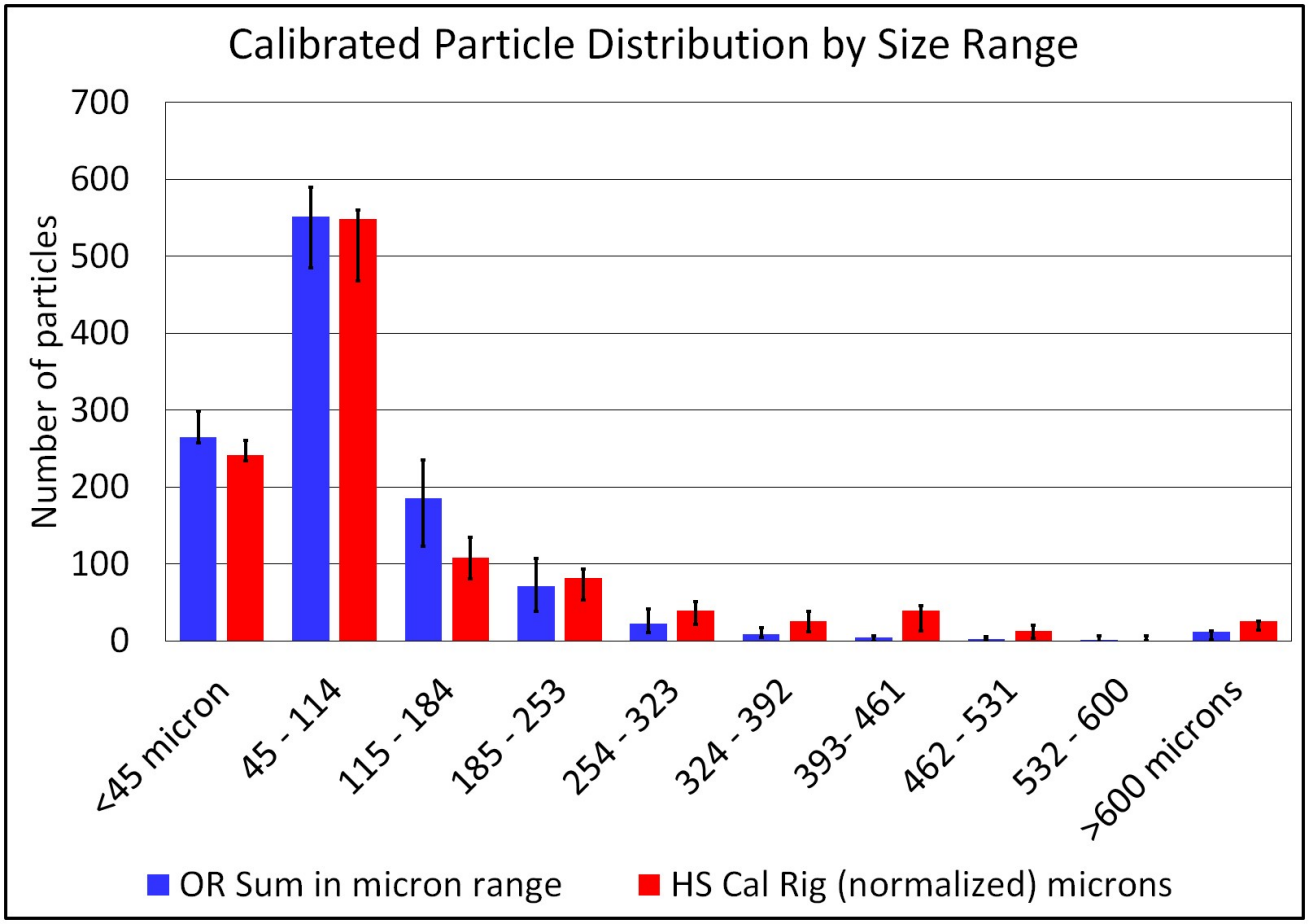
Histogram of particles parsed into ten ranges for improved match.

The best fit result was a ten-bin parsing of the ranges (as seen in Fig 12, a maximum range (overflow bin) collecting all particles above 600 microns, and a minimum range (underflow bin) collecting all particles less than 45 microns.

The resulting data fit yielded a correlation mapping between the effective diameters measured with the OR System and the calibrated diameters measured with shadow imaging setup such that

$$\begin{array}{c} d(B)=13.9*B-168.7 \end{array}$$ (4)

where d is the particle diameter in microns and B is the measured effective diameter in pixels.

Due to the previously mentioned linear resolution limits, this relationship fails below a measured effective diameter of 13.9 pixels and thus a linear fit to zero was used below this point. The final conversion function was

$$\begin{array}{c} d\left(B\right)=\{\begin{array}{c} 13.9*B-168.7,B>14.2\\ 3.2374*B,B\leq 14.2 \end{array} \end{array}$$ (5)

## Error and uncertainty in measurement

This will be a systematic uncertainty analysis since there is not enough data to make a statistical uncertainty analysis. That is, it will be on the systematic properties of the measurements and the errors in how they were obtained. There are two main sources of error which include uncertainty in diameter measurement in the shadow measurement and uncertainty in diameter measurement in the OR system. Since these measurements are not connected physically, they are independent sources.

### OR system uncertainty

The OR system has a larger uncertainty due to the non-linear scaling of laser scattering from the particles. Using the “Out of Focus” compensation method described above minimizes this error. Nevertheless, there are still errors in the determination of the boundary within blurry edges and, if the determination is taken to a maximum or minimum particle edge size, the pixel diameter will vary. Several particle images were evaluated to determine the maximum variability in size, and the result is an uncertainty that grows with pixel size (and therefore particle size in microns) but as a percentage, it reduces as seen in Eq 6.

$$\begin{array}{c} U_{\text{OR}}\left(n\right)=\left[0.0518*d\left(n\right)\right]+0.9286 \end{array}$$ (6)

Where *U*_*OR*_(*n*) is the maximum particle (*n*) diameter uncertainty in microns for an OR System particle and *d*(*n*) is the measured diameter for that particle (*n*).

### Shadow method uncertainty

Measuring particle sizes using the shadow method (used in the calibration system) is inherently more accurate; however, it does suffer from out of focus particles due to the very shallow depth of field. That is, if a particle is more than ± 3mm from the focus depth, it becomes blurry. For these particles, the fuzzy boundary is a minimum of one pixel in diameter and grows with particle size. It was found that maximum particle uncertainty followed a 3% trend with size.

$$\begin{array}{c} U_{\text{cal}}\left(n\right)=0.03*d\left(n\right) \end{array}$$ (7)

Where *U*_*cal*_(*n*) is the uncertainty in any particle (*n*) size measurement *d*(*n*) in microns.

### Total measurement uncertainty

For two independent measurement uncertainties, the total uncertainty is the square root of the sum of the squares of the measurement uncertainties as seen in Eq 8.

$$\begin{array}{c} U_{\text{Tot}}\left(n\right)=\sqrt{U_{cal}{\left(n\right)}^{2}+U_{OR}{\left(n\right)}^{2}} \end{array}$$ (8)

Which equates to

$$\begin{array}{c} U_{\text{Tot}}\left(n\right)=\sqrt{{\left(0.03*d\left(n\right)\right)}^{2}+{\left(0.518*d\left(n\right)+0.9826\right)}^{2}} \end{array}$$ (9)

and is simplified to

$$\begin{array}{c} U_{\text{Tot}}\left(n\right)=\sqrt{{\left(0.927*d\left(n\right)\right)}^{2}+0.096*d\left(n\right)+0.861} \end{array}$$ (10)

Although this non-linear uncertainty varies with diameter, it is approximately 6% of diameter. It varies from 6.4% on small particles to just under 6% at 600 μm (or 3 microns for 45μm particles and 36 μm for 600 μm particles).

### Histogram uncertainty

For calibration, each particle generator produces particles in a spectrum of sizes and numbers and the shadow method and OR System method are used to measure every particle from every generator. The particles from each method are gathered into separate histograms. The shadow method pixel values are converted to micron dimensions. To compensate for different total numbers between the two methods, a gain multiplier is placed on the shadow method bin sizes. The correlation is done by adjusting the side scatter method “gain and offset” of pixel size to micron size to achieve a histogram matching the shadow method.

The uncertainty for a bin in the histogram for the shadow (calibration) method is then broken into two parts. The lower uncertainty range in a histogram bin is calculated from the percentage of particles in a bin that are within range of being in a higher or lower bin (and leaving the bin). The upper uncertainty range is calculated form the percentage of particles that could enter a bin due to being within an uncertainty percentage of a bin boundary outside the bin of interest.

Several factors are considered. The bottom and top bins can only take and lose bins above and below respectively. If the lower uncertainty is greater than the number in the bin, the lower uncertainty is the total bin number.

## Results and discussion

This describes the development of a system to measure both the size and quantity of particles exiting a patient in an operating room. It was performed as a result of interest from surgeons in an operating room on the transmission of the Covid-19 Virus. To achieve this goal, two systems were developed, a calibration system and an OR system. The OR system uses side scattering of laser light and the result is a wide range of particle size measurement. For our measurements, the calibration range was from below 40 microns to over 600 microns.

To work in an operating room, the OR system included a ∼400mm dark chamber with a 75mm diameter entrance aperture through which the particles travelled. A sheet of laser light was focused behind this aperture to generate the side scatter of light. The chamber was designed asymmetrically and reversible to fit close to both the left and right sides of the patient. Since the first goal was to measure during a thoracostomy, the dark chamber was also designed to fit tightly under the armpit which placed the aperture close to one end of the dark chamber. To prevent strong reflections, an effective light trap was added (on both sides of the chamber). To minimize pre-existing ambient particles prior to measurements, a fan blowing through a small HEPA filter was installed to purge the internal air. To minimize the flow restriction, the chamber was made larger than expected (64 liters) and (during measurements), the purge fan was reversed to draw air in through the aperture.

The calibration system employed the measurement of shadows; however, this was more difficult than expected. To see the tiny particle, the camera had to be configured with very high physical pixel resolution and the velocity of the particles across the image field of view was such that the image acquisition speed had to be very high. However, this increase in speed reduced the camera resolution and so a compromise was found around 10,000 frames per second. Given the velocity of image FOV, the exposure time was shortened to 23 *μ*s. This short exposure time required the use of a powerful lamp to adequately illuminate the field of view. The system worked well and measurements were easy to interpret; however, some particles were slightly blurry and a technique was developed to determine their size.

In order to avoid the cost and complexity of devices like a vibrating orifice generator, particles were produced using standard hand-held spray bottles. In order to obtain a wide range of particle sizes, several spray nozzles were modified using jet drills and the five nozzles varied from 0.46 to 1.00 mm diameter. The sprays from these nozzles were recorded by both the shadow calibration system and the OR system and compared.

The authors employed inexpensive and/or readily available components that were easily accessible and likely to exist in many research institutions and hospitals. As an example, superior high-resolution, high-speed cameras are available; however, most contemporary tablets available today have an amazing ability to capture video at high resolution and proved to be sufficient for this application.

There are several areas where this method could be improved for long term use. The first of which is the use of commercially available software packages that could automate the particle size measurement. This would remove some of the potential errors associated with manual processing and improve the ability to perform measurement error analysis. Additionally, a more appropriate, scientific camera could remove potential variability due to the tablet’s limited lens control and automatic image compression. In combination with the camera and software, an improved method of generating particles with more controlled sizes—such as with a vibrating orifice generator—would improve the precision of resulting calibration.

Finally, improving the design to make a modular or compressed enclosure/laser to allow a better fit to a patient which could be supported from an overhead arm as used in ORs would make the system more user-friendly.

## Summary

We present a system for in situ measurement of airborne particles in a large field of view and include a calibration method for quantifying particle size. The calibration method, equipment and system design—which allows for use in specialized AGPs—is described.

The optical measurement system (OR System) uses a laser side-scatter technique to yield measurements of airborne particles in the range of less than 25 μm to over 600 μm across a relatively large field of view of ∼75 mm. A laser light sheet is placed across the path of exhaust particles from a patient and imaged with a standard tablet camera. Side-scattered light eliminates the interference issues related to other techniques such as diverting the particle flow through a pipe.

A correlation and calibration method is presented whereby controlled particle sizes were generated using a range of simple spray nozzles and measured with a shadow imaging system. The shadow imaging system was able to capture the small (down to 20 μm) and fast (up to 10 m/s) particles with a high-speed camera at 10,000 fps and exposure time of 23 μs, resulting in a field of view of 7.9 x 7.1 mm. The scaled/calibrated particle diameters were determined directly from their shadows. The same particle generators were then measured using the OR System which employs laser side-scatter imaging and a correlation is made to map the calibrated diameters from the shadow imaging system.

An aluminum frame and foam-board enclosure were constructed to position and align the system components while safely containing the laser light. The mobile OR System was developed for use in a hospital environment and the configuration was aligned with the needs of measuring airborne bodily exhaust during AGPs such as thoracotomies/thoracostomies and tracheotomies. To meet these requirements, the system was designed to be symmetric and reversible in order for the measurement aperture to be placed in proper proximity to the patient’s body.

## Supporting information

S1 File Summary calibration measurements. (XLSX)
